# Acupuncture is a feasible treatment for post-thoracotomy pain: results of a prospective pilot trial

**DOI:** 10.1186/1471-2253-6-5

**Published:** 2006-05-03

**Authors:** Andrew J Vickers, Valerie W Rusch, Vivek T Malhotra, Robert J Downey, Barrie R Cassileth

**Affiliations:** 1 Department of Medicine, Memorial Sloan-Kettering Cancer Center, New York, New York, USA; 2 Department of Epidemiology and Biostatistics, Memorial Sloan-Kettering Cancer Center, New York, New York, USA; 3 Department of Surgery, Memorial Sloan-Kettering Cancer Center, New York, New York, USA; 4 Department of Anesthesiology, Memorial Sloan-Kettering Cancer Center, New York, New York, USA

## Abstract

**Background:**

Thoracotomy is associated with severe pain that may persist for years. Acupuncture is a complementary therapy with a proven role in pain control. A randomized trial showed that acupuncture was effective in controlling pain after abdominal surgery, but the efficacy of this technique for the treatment of thoracotomy pain has not been established. We developed a novel technique for convenient application of acupuncture to patients undergoing thoracotomy, and in a Phase II trial evaluated the safety of this intervention and the feasibility of doing a randomized trial.

**Methods:**

Adult patients scheduled for unilateral thoracotomy with preoperative epidural catheter placement received acupuncture immediately prior to surgery. Eighteen semi-permanent intradermal needles were inserted on either side of the spine, and four were inserted in the legs and auricles. Needles were removed after four weeks. Using a numerical rating scale, pain was measured on the first five postoperative days. After discharge, pain was assessed using the Brief Pain Inventory at 7, 30, 60 and 90 days.

**Results:**

Thirty-six patients were treated with acupuncture. Of these, 25, 23, and 22 patients provided data at 30, 60, and 90 days, respectively. The intervention was well tolerated by patients with only one minor and transient adverse event of skin ulceration.

**Conclusion:**

The rate of data completion met our predefined criterion for determining a randomized trial to be feasible (at least 75% of patients tolerated the intervention and provided evaluable data). This novel intervention is acceptable to patients undergoing thoracotomy and does not interfere with standard preoperative care. There was no evidence of important adverse events. We are now testing the hypothesis that acupuncture significantly adds to standard perioperative pain management in a randomized trial.

## Background

Thoracotomy is a common procedure employed for primary lung cancer, with almost 30,000 performed annually in the United States (Source: Agency for Healthcare Research and Quality). The procedure is extensive and can be associated with severe pain in patients already compromised by numerous other co-morbidities such as chronic obstructive pulmonary disease. Pain that limits respiratory function can have devastating consequences on post-operative recovery; consequently, extensive measures such as epidural catheters, local anesthetic infiltration and nonsteroidal-anti-inflammatory drugs (NSAIDs), are used to maximize analgesia and minimize side effects. [1,2]. Acupuncture represents a modality providing analgesia with a paucity of side effects; however, acupuncture needles can potentially overlap with the surgical field and also hamper epidural catheter placement, making acupuncture impractical. We undertook a single-arm, open-label, clinical trial to assess: 1) the feasibility of employing pre-operative acupuncture needles near the operative field for analgesia after thoracotomy, 2) the acceptance by patient's of a non-traditional technique after receiving a diagnosis as ominous as cancer, 3) the percentage of patients providing evaluable pain data at the first post-operative visit (approximately 30 days post surgery), 4) the occurrence of any adverse effects, 5) to determine the optimal primary outcome and timepoint for the randomized trial, 6) to provide data to determine sample size calculations. With respect to the primary aim, the trial was considered "feasible" if at least 75% of patients tolerated the intervention; if the intervention did not interfere with surgery and routine post-operative care; if at least 75% of patients provided evaluable data at the first post-operative visit; and, if reported adverse events were acceptable. Furthermore, accrual needed to be sufficiently rapid so that, after appropriate sample size calculations, a randomized trial could be completed in less than two years.

## Methods

The study was approved by the Institutional Review Board (IRB) at Memorial Sloan-Kettering Cancer Center in accordance with an assurance filed with and approved by the Department of Health and Human Services. Written informed consent was obtained from each participant.

### Eligibility criteria

We recruited adult patients at Memorial Sloan-Kettering Cancer Center who were scheduled to receive a unilateral, posterolateral thoracotomy with analgesia using an epidural catheter.

Patients excluded from the study were those scheduled to undergo "hemiclamshell" or "clamshell" thoracotomy, extrapleural pneumonectomy, or esophagectomy. Patients who had received acupuncture within the previous six weeks, or who had heart valve dysfunction (a contraindication to the use of intradermal acupuncture needles) were excluded. Patients with a coagulopathy precluding insertion of an epidural catheter were also ineligible. All of the patients entered on this study were operated on by a single surgeon (VR), which allowed for uniformity of surgical technique and perioperative care.

#### Treatment

Epidural catheters were placed in all patients in a pre-operative holding area with all patients in the sitting position. An iodine antiseptic was used to sterilize both the field for epidural placement and acupuncture placement. An 18 gauge Tuohy needle was used identify the epidural space, typically at the level of T6–T12. After placement of the epidural catheter, a test dose of lidocaine 1.5% and epinephrine 1:200,000 in a volume of 2–3 ml was used to identify accidental intrathecal or intravascular replacement. If either occurred, the epidural catheter was either replaced or the procedure aborted at the discretion of the physician.

**Figure 1:**
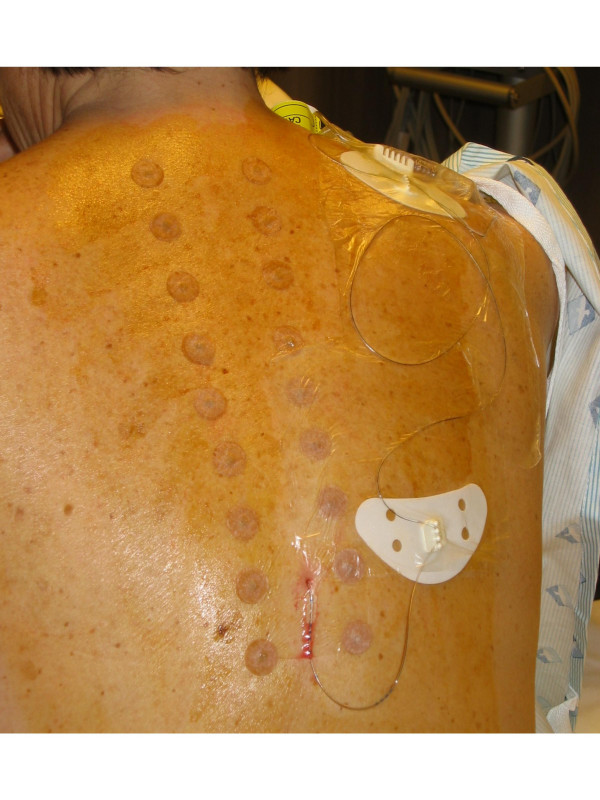
Intradermal acupunctures needles used in this study.

Eighteen semi-permanent intradermal acupuncture needles were inserted at points "BL12" to "BL19" and extra point Wei Guan Xia Shu. These points are approximately 2.5 cm lateral to the lower border of spinous process of the T2 – 10 vertebrae immediately after placement of the epidural catheter (see Figure 1). Subsequently, both the epidural catheter and acupuncture needles were covered simultaneously by an occlusive Tegaderm™ dressing. In addition, one needle was placed in each leg at the "ST36" point and one in each auricle at the "Shenmen" point. This point prescription is based on that of Kotani et al.[3], who reported a randomized trial showing that intradermal needles at these points reduced pain compared to placebo. We added the "Shenmen" and "ST36" points on the grounds that these are commonly used to treat pain.

We initially used "Japanese" style needles, as described by Kotani et al.[3], but found these time-consuming to apply and switched to stainless steel AcuMedic intradermal needles (AcuMedic Ltd., London, UK). These are thumbtack-shaped and consist of a 2 mm × 0.28 mm acupuncture needle attached to a metal ring embedded in surgical tape.

Patients underwent balanced general anesthesia primarily with inhalational agents (typically Isoflurane). In addition, epidural analgesia was initiated in the operating room using a continuous infusion of fentanyl (10 mcg/cc) and bupivacaine (0.05%) at a fixed rate of 4 milliliters per hour. Hydromorphone or morphine were chosen for the epidural instead of fentanyl if an adverse reaction precluded the use of the latter. Of the evaluable patients, ten were initiated on fentanyl and fifteen patients on hydromorphone. In addition analgesia was supplemented with intravenous narcotics as needed. Unless contraindicated, all patients received ketorolac 15–30 mg intravenously prior to closure and in the post-operative period as needed for up to 3 days. If a patient had persistent side effects with subjective complaints of unacceptable analgesia, the epidural opioid was changed to either hydromorphone or morphine or removed entirely; nine patients received more than one drug. Persistent hypotension was ultimately treated by removal of the epidural local anesthetic.

The epidural catheter and acupuncture needles were both removed after removal of the chest tube, typically 3 to 7 days post surgery. Patients were given oral medications (typically oxycodone with acetaminophen) as needed to manage any ongoing pain. All needles were replaced after the epidural catheter was removed, typically 24 to 48 hours before discharge from the hospital. No restrictions were placed on patients' activity level or showering. Patients were asked to remove the leg and auricular needles a week after discharge. Needles on the back were removed by an acupuncturist at the first follow-up visit, usually 3 to 4 weeks postoperatively. Patients who remained in pain at this time were offered further acupuncture treatment consisting of stainless steel, 15 mm × 0.16 mm Seirin auricular needles placed bilaterally for 20–25 minutes at the "Shenmen", "brain" and "adrenal" points in the ear. In addition, up to two needles were placed at auricular points corresponding to the sites where patients were experiencing most pain (e.g. chest, abdomen or ribs). Brief manual stimulation was used immediately after insertion. Practitioners attempted to achieve "de qi", but this was assessed by needle sensation rather than questioning of the patient. Following this treatment, acupressure metal balls were applied at the same points, and patients were taught to press each point for two minutes twice a day. They were also instructed to press on the points if they experienced exacerbation of pain. The metal balls typically remained in place for 4–7 days. These were used in preference to intradermal needles for this stage of treatment, when there is no further contact with an acupuncturist, as they do not penetrate the skin and there is no risk of infection were the patient to forget to remove them.

### Acupuncture quality control

Immediately after each needle insertion, acupuncturists completed an audit sheet verifying the acupuncture points used. These records were routinely reviewed and acupuncturists were found to have followed the acupuncture point prescription. Study acupuncturists are certified by the National Certification Commission for Acupuncture and Oriental Medicine (NCCAOM) and are licensed to practice acupuncture in New York State. They have used acupuncture in clinical practice for 3 – 25 years. All were employees of the Integrative Medicine Service at Memorial Sloan-Kettering Cancer Center.

### Primary outcome

We specified that we would consider the trial feasible if at least 75% of patients tolerated the intervention; if the intervention did not interfere with surgery and routine post-operative care; if at least 75% of patients provided evaluable data at the first post-operative visit; and, if reported adverse events were acceptable. We also wanted to ensure that accrual was sufficiently rapid so that, after appropriate sample size calculations, a randomized trial could be completed in less than two years.

### Pain assessment

Pain in the immediate postoperative period was assessed by a 0–10 point numerical rating scale (NRS) marked "no pain" at one end and "worst pain" at the other. Patients were evaluated between 4:00 pm and 6:30 pm on the evening of their surgery, at least two hours after leaving the Operating Room. Those not leaving by 4.30 pm had their first pain evaluation on the following day. On the first postoperative day, patients were evaluated in the morning and late afternoon. The first pain evaluation assessed pain at rest, on movement and on coughing "since you woke up from your operation." Subsequent evaluations (post-operative days 2, 3, 4 and 5) evaluated pain at rest, on movement and with coughing since the previous evaluation.

After discharge from the hospital, pain was assessed 1 week from removal of the epidural catheter (11–18 days after surgery) and again approximately 30, 60 and 90 days postoperatively. The 11–18 day follow-up was conducted by a research study assistant by telephone; all others were conducted when patients returned for routine post-discharge appointments. Pain was evaluated using the Brief Pain Inventory (BPI), a validated pain measurement tool that measures both the intensity of pain (sensory dimension) and interference of pain in the patient's life (reactive dimension) [4]. Patients were also asked to report if they experienced any pain which they attributed to their operation. The number of needles remaining in place at the postoperative visit was recorded.

### Data analysis

Data were analyzed using Stata 8.2 (Stata Corp., College Station, TX). We chose an actual sample of 25 patients. Our stopping rule was for the trial to cease once 25 patients had provided data at the 30 day follow-up.

## Results

### Patient accrual and adverse events

**Figure 2:**
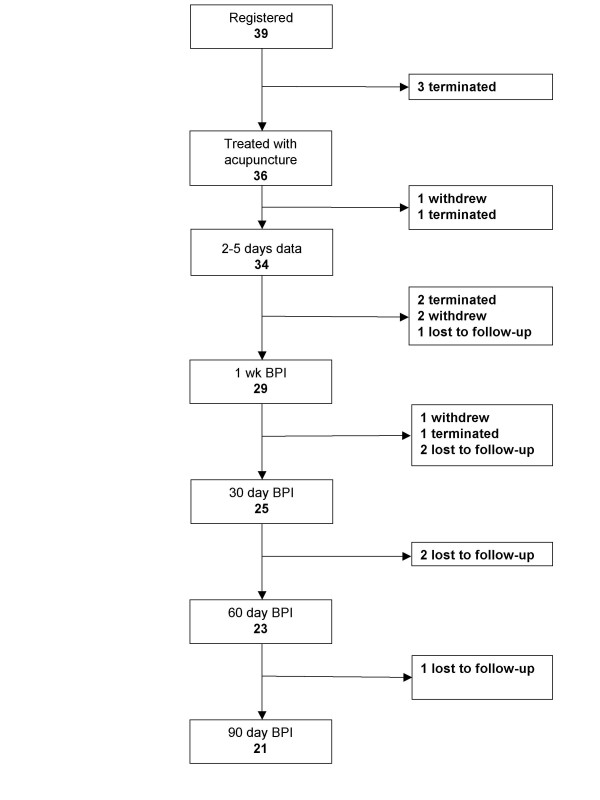
Participant flow through the trial.

39 patients were accrued between August 2002 and September 2003. Participant flow is shown in Figure 2. Accrual was limited for this pilot study because patients were accrued only from a single clinician (VR) for operation on a single day of the week. Patient acceptance was very good, with fewer than five patients refusing participation in the trial.

Twenty-five patients (64%) provided evaluable pain data at the first post-operative visit. Seven patients were terminated by investigators for protocol reasons such as the epidural catheter not being used, or removed early due to inadequate analgesia. None of these terminations would occur in a randomized trial due to the "intent-to-treat" principle. Of the patients who were not withdrawn, 25 of 32 provided evaluable data, a 78% data completion rate, which meets our predefined criterion for doing a subsequent randomized trial. Three patients could not be contacted and four withdrew, of whom one expressed distrust of hospital staff, one reported no pain, one did not want to deal with needles after discharge and one withdrew without giving a reason.

Table 1 provides basic demographic information about study participants. Most patients (62%) were women in their 60 s, undergoing lobectomy for primary lung cancer. More men and patients undergoing procedures other than lobectomy dropped out of the study.

**Table 1:** Baseline patient data

	Completed day 30 data (n = 25)	Did not provide day 30 data (n = 14)	All
Age			
less than 60	8 (32%)	3 (21%)	11 (28%)
60–70	12 (48%)	8 (57%)	20 (51%)
greater than 70	5 (20%)	3 (21%)	8 (21%)
Female	18 (72%)	6 (43%)	24 (62%)
Diagnosis			
Primary lung cancer	23 (92%)	13 (93%)	36 (92%)
Lung metastases	2 (8%)	1 (7%)	3 (8%)
Procedure			
Exploration only	2 (8%)	2 (14%)	4 (10%)
Pneumonectomy	0 (0%)	2 (14%)	2 (5%)
Lobectomy	18 (72%)	7 (50%)	19 (64%)
Wedge resection	5 (20%)	3 (22%)	8 (21%)

None of the remaining patients requested or required removal of the acupuncture needles before discharge. Of the 25 patients with 30-day data, there were no data on needle retention for two: one patient received follow-up care at a different institution and one was discharged before reinsertion of needles. Of the 23 remaining patients, 12 (52%) either retained 16 or more needles out of 18; 4 patients (18%) retained fewer than half the needles and were thus defined as not tolerating the intervention. If we include the patient who withdrew because of complaints about the needles, 19 out of 24 patients (79%) retained more than half the applied needles at 30 days.

Treatment was very well tolerated, with no patient complaining of discomfort from the needles or the Tegaderm. Seven adverse events were reported to the IRB of which six had clearly had no relationship to acupuncture, such as gastrointestinal infection. One patient was noted to have a 1 cm diameter superficial skin ulcer at 30 day follow-up. This was under the Tegaderm but away from the acupuncture needles. The Tegaderm was removed, the ulcerated area cleaned and Bacitracin ointment applied. The ulceration healed within four days.

After changing from the "Japanese" style to the Acumedic needles (see Methods), we found that application of needles in the pre-operative period was rapid, and did not interfere with routine care.

### Results of pain assessment

Pain data for the immediate postoperative period are shown in table 2; table 3 shows data for post-discharge follow-ups. Although data from this single arm feasibility trial does not permit assessment of the efficacy of acupuncture in the management of post-thoracotomy pain, the data show decreasing severity of pain over time. The design of this trial is unable to demonstrate that this effect is from acupuncture as the primary aim is the feasibility of performing acupuncture. Twenty-two of 25 patients who provided data at day 30 also provided data at day 90, suggesting the feasibility of obtaining long-term data. The apparently higher pain scores at day 90 compared to day 60 probably do not indicate increasing pain as it is well within typical statistical variation.

**Table 3:** Pain scores (BPI) after discharge vs. post-operative day. Results are given as mean (SD). Only patients reporting use of analgesics were asked to report relief. *(Lower numbers indicate less pain)*

Day	Pain from operation	BPI total	BPI pain intensity	BPI pain interference	BPI relief	Analgesic use
7	28 (97%)	n = 29: 3.88 (1.91)	n = 29: 3.6 (1.52)	n = 28: 3.94 (2.42)	n = 24: 7.25 (2.27)	26/29 (90%)
30	21 (84%)	n = 25: 2.92 (1.79)	n = 25: 2.62 (1.49)	n = 25: 3.09 (2.25)	n = 20: 7.4 (2.14)	19/25 (76%)
60	16 (70%)	n = 23: 1.75 (1.96)	n = 23: 1.72 (1.76)	n = 23: 1.78 (2.21)	n = 8: 7 (2.2)	8/23 (35%)
90	11 (50%)	n = 22: 2.17 (2.36)	n = 22: 2.13 (1.98)	n = 22: 2.18 (2.79)	n = 9: 7.22 (1.72)	10/22 (45%)

## Discussion

There are multiple possible sources of post thoracotomy pain, including the skin incision, muscle division, rib fracture or resection, costochondral dislocation, neuroma formation and, less commonly, infection or tumor recurrence. Acute pain in post-operative period may lead to chronic pain characterized as "the post-thoracotomy pain syndrome."[5] This is defined as an aching or burning pain that persists or recurs along a thoracotomy scar two months or more after surgery. Several studies estimate the prevalence of short-term post thoracotomy pain at 50% to 80% [6,7]. Approximately 5% of all patients undergoing thoracotomy suffer severe or incapacitating pain that interferes significantly with their daily activities. After an initial decline, there is little evidence demonstrating an appreciable diminishment of post thoracotomy pain over time. Although one study reported the prevalence of pain falling from 80% at 3 months post-surgery to 61% at 1 year[8] a longitudinal study examined patients 1 to 5 years post-surgery and found stable pain intensity over the course of follow-up, with approximately 50% patients reporting some level of pain[9].

Absolute control of pain following thoracotomy using traditional analgesics can be associated with many side-effects, some of which (like sedation) can have devastating consequences on patients with co-morbidities such as lung disease and heart disease. Acupuncture is a well-known complementary treatment for pain [10] with few to no side effects, and data from recent meta-analyses suggest that it can relieve both acute and chronic pain[11]. In this study we employed it pre-operatively in a pre-emptive fashion because of ease of placement compared to post-operative placement.

Recent data suggest that acupuncture is effective in relieving pain following abdominal surgery[3]. In that study, 185 patients scheduled for surgery were randomized to receive true or placebo acupuncture. In the true acupuncture group, 5 mm semi-permanent acupuncture needles were inserted preoperatively at 14 points in the back and fixed in place with surgical tape. Patients assigned to the control group had needles placed perpendicularly so that they did not penetrate the skin. Pain control was superior in the acupuncture group; the percentage of patients with moderate or severe pain at rest on the day immediately following surgery was 72% in the control group but only 47% in those receiving true acupuncture. Patients in the acupuncture group also received approximately 25% less morphine during the first four postoperative days than did placebo patients.

Although these data are promising, we were concerned that the severity and duration of pain reported after abdominal surgery was somewhat less than is typical after thoracotomy. We felt it would be necessary to provide acupuncture stimulation over a longer period of time than attempted by Kotani et al.[3] and hypothesized that 30 days would be ideal. Therefore, we inserted needles immediately preoperatively, changed them before discharge from the hospital and then did not remove them until the first follow-up visit approximately 3 to 4 weeks postoperatively. This entails needles being retained for three weeks or more.

In this study, we demonstrated that a novel acupuncture intervention was acceptable to staff and patients (aim 2). We found no evidence of important adverse effects (aim 4). To our knowledge, this is the first reported clinical trial to evaluate acupuncture in the management of post-thoracotomy pain. The acupuncture procedure took only five minutes to complete and did not interfere with standard preoperative care (aim 1), such as placement of the epidural catheter. Although fewer patients than intended provided follow-up data, the predicted rate of data completion for the randomized trial met our predefined criterion (aim 3). We propose that the BPI pain intensity score be used as our primary endpoint as this had the lowest coefficient of variation (aim 5). We also propose 30 day follow-up as the primary endpoint (aim 5). Although we are also interested in chronic post-thoracotomy pain, this ideal has to be considered against the potential of increased patient drop-out over time. From these and others' data [12], pain at 30 days appears reasonably predictive of longer-term outcome.

Accrual was relatively slow because this pilot of a novel technique accrued patients only from a single clinician, a maximum of one day each week. To determine sample size for the trial (aim 6) we used the mean BPI pain intensity score at the 30 day follow-up (2.6) and took the upper 75th centile of the standard deviation (1.63). We assumed a minimum, clinically significant difference of 25%, i.e., that BPI scores will be 25% lower in the acupuncture group compared to placebo. This gives a hypothesized score in the control group close to 3.6. This is approximately equivalent to the expected incidence of moderate or severe pain (defined as pain score = 4) being 20% in the acupuncture group and 40% in controls. With a 5% alpha and a power of 80%, 52 evaluable patients per group are required. Allowing for 25% drop-out, we anticipate accruing approximately 140 patients. The Thoracic Service at MSKCC performs an average of 550 thoracotomies annually that would meet study eligibility criteria. We would need to accrue 13% of patients to meet our target sample within two years, or 9% to meet accrual targets within three years. Both targets seem highly feasible. The randomized trial has been approved by our Institutional Review Board and has been initiated The results of this randomized trial will determine whether acupuncture plus standard continuous epidermal or intravenous analgesia is superior to standard systemic analgesia alone.

## Conclusion

The rate of data completion met our predefined criterion for determining a randomized trial to be feasible (at least 75% of patients tolerated the intervention and provided evaluable data). This novel intervention is acceptable to patients undergoing thoracotomy and does not interfere with standard preoperative care. There was no evidence of important adverse events. We are now testing the hypothesis that acupuncture significantly adds to standard perioperative pain management in a randomized trial.

## Conflicts of interest

The author(s) declare that they have no competing interests.

## Authors' contributions

All authors contributed to study design. AV was responsible for methodologic and statistical aspects; BC advised on clinical aspects of implementing complementary therapies; VR and RD on surgical aspects of thoracotomy and VM on clinical pain control. Data analysis was undertaken by AV. All authors contributed to data interpretation, helped revise the manuscript for important intellectual content and approved the final version.

**Table 2:** Pain scores (VAS) in the immediate postoperative period versus post-operative day. Results are given as mean (SD). Not all patients reported cough. *(Lower numbers indicate less pain)*

Day	Pain at rest	Pain on movement	Pain on cough
0	n = 8: 5 (2.39)	n = 7: 5.86 (2.97)	n = 4: 5.75 (4.35)
1	n = 34: 2.5 (2.15)	n = 34: 4.38 (2.74)	N = 31: 5.08 (2.97)
2	n = 32: 1.97 (1.86)	n = 32: 3.66 (2.32)	N = 22: 5.68 (2.25)
3	n = 29: 2.32 (2.25)	n = 29: 4.48 (2.44)	N = 20: 5.55 (2.63)
4	n = 15: 2.00 (2.00)	n = 15: 3.20 (2.11)	n = 8: 3.63 (3.07)
5	n = 9: 1.78 (2.22)	n = 9: 4.00 (2.45)	n = 5: 5.20 (2.77)

## Pre-publication history

The pre-publication history for this paper can be accessed here:


